# The effect of maxillary molar distalization with clear aligner: a 4D finite-element study with staging simulation

**DOI:** 10.1186/s40510-023-00468-1

**Published:** 2023-05-15

**Authors:** Bochun Mao, Yajing Tian, Yujia Xiao, Jing Li, Yanheng Zhou

**Affiliations:** Department of Orthodontics, Peking University School and Hospital of Stomatology & National Center for Stomatology & National Clinical Research Center for Oral Diseases & National Engineering Research Center of Oral Biomaterials and Digital Medical Devices & Beijing Key Laboratory of Digital Stomatology & Research Center of Engineering and Technology for Computerized Dentistry Ministry of Health, No.22 Zhongguancun South Avenue, Haidian District, Beijing, 100081 People’s Republic of China

**Keywords:** Finite-element method, Long-term simulation, Clear aligner, Molar distalization

## Abstract

**Introduction:**

Long-term simulation of tooth movement is crucial for clear aligner (CA) treatment. This study aimed to investigate the effect of maxillary molar distalization with CA via an automatic staging simulation.

**Method:**

A finite-element method (FEM) model of maxillary dentition, periodontal ligaments, attachments, and corresponding CA was established, and a prescribed 2-mm distalization with 0.1 mm each step of the second molar was simulated. The long-term tooth movement under orthodontic force was simulated with an iterative computation method. The morphologic changes of CA during staging were simulated with the thermal expansion method.

**Results:**

Twenty steps of molar distalization were simulated. Significant distal tilting of the second molar was revealed, along with the proclination of anterior teeth, which caused the ‘reversed bow effect’. For the second molar, 4.63°distal tilting at the 20th step was revealed. The intrusion of the incisors and the second molar were 0.43 mm, 0.39 mm, and 0.45 mm, respectively, at step 20. All the anterior teeth showed a proclination of approximately 1.41°–2.01° at the 20th step. The expression rate of the designed distalization of the second molar was relatively low (approximately 68%) compared to the high efficacy of interdental space opening between molars with CA (approximately 89%).

**Conclusion:**

A novel method of simulating long-term molar distalization with CA with FEM was developed. The FEM results suggested distal tilting of the second molar and the proclination of anterior teeth during the molar distalization.

## Introduction

The unsatisfactory predictability and accuracy of clear aligner (CA) treatments have been long-debated issues for decades [1, 2]. Unlike fixed appliances, the mechanical essence of CAs, i.e., the continuously changing contact relationship, makes it difficult for researchers to determine the acting position or moments of forces of CAs. Among all designed teeth movement with CA, molar distalization has the highest expression rate of 88% [3], which makes molar distalization a preferred treatment strategy for patients to acquire a 2–3-mm arch space and Class I molar relationship. However, the main side effect of molar distalization is the loss of anchorage of anterior teeth, which manifests as flared incisors and the protrusion of lips [4, 5]. Other side effects including intrusion, distal tipping, and distal rotation of molars, have also been revealed [6, 7].

With a clear biomechanical understanding of CAs, orthodontists can achieve safe, predictable, and stable treatment outcomes. The finite-element method (FEM) is a numerical method for studying the stress distribution and distortion of any given geometry. The FEM has been widely used in investigating the biomechanical behavior of CAs, including the different CA thicknesses, attachment designs, elastic designs and so on [8–10].

However, for current biomechanical studies based on FEM, most studies were limited to analyses of the initial mechanical condition, displacement in the periodontal ligament (PDL) and other models, which cannot provide adequate information for long-term tooth movement during orthodontic treatment. To solve this problem, in recent years, researchers have carried out ‘four-dimensional (4D)’ FEM, which takes the biomechanical response as another dimension, and have revealed several iterative computation methods to simulate tooth movement during orthodontic treatment [11–15]. The 4D FEM was first introduced into the medical field to simulate the thoracic complex dynamic impedance change, and many clinically significant results were gained with the study of heartbeat and aortic wave propagation [16, 17]. With 4D FEM, which could simulate constant changing interrelationships among all components within a simulation model, orthodontists can gain more significant results for long-term orthodontic treatment.

However, all the current iterative computation methods for long-term orthodontic simulation could only be used in the simulation of fixed appliances since there is no need for model remodeling during the iteration; therefore, it is easier to perform than CA treatment simulation. Thus, for CA simulation, all currently published studies are limited to the analysis of initial displacement when wearing CAs, which provides inadequate results for clinical reference.

To our knowledge, no previous study has considered long-term tooth movement simulation for CA treatment with multiple steps of CAs during staging. Thus, this study aimed to simulate long-term maxillary second molar distalization with CAs by ① an automatic CA iterative remodeling method, ② an iterative computation method of tooth movement simulation considering actual treatment time, and ③ the combination of the above two iterative computation methods.

## Method

**Fig. 1:**
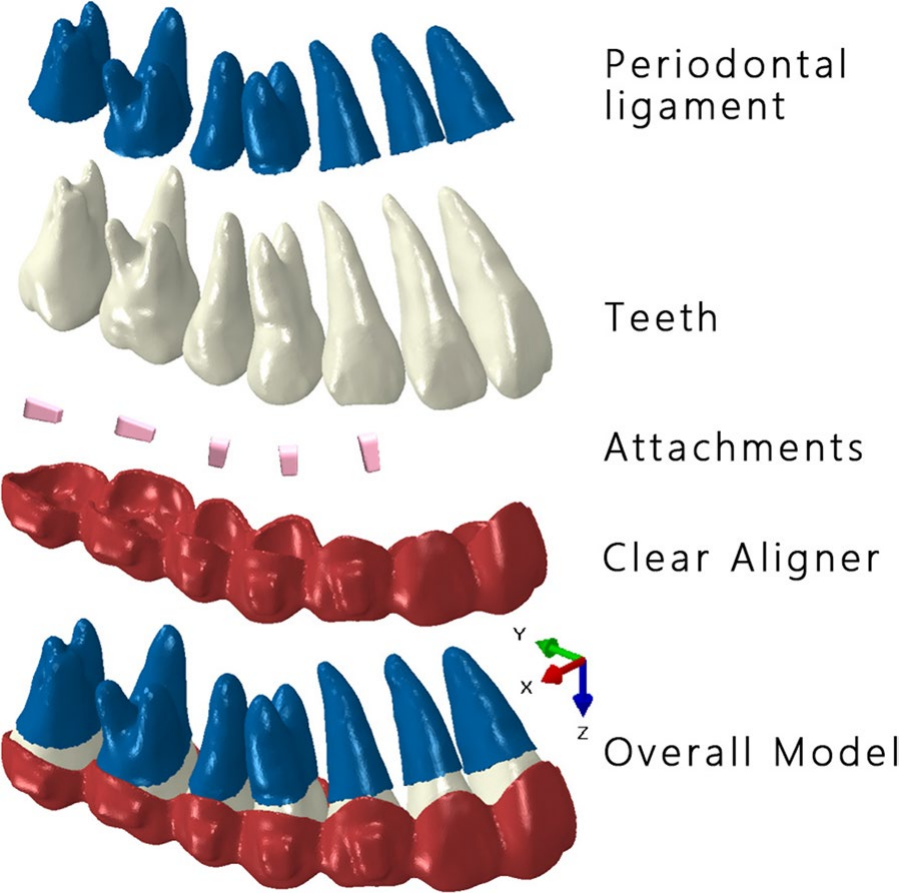
Components of the model

This research was approved by the Institutional Review Board of Peking University School and Hospital of Stomatology (PKUSSIRB-202059154), and all experiments were performed in accordance with relevant guidelines and regulations. A digital dental cast, scanned with a desktop scanner (3shape R900 model scanner, 3shape, Copenhagen, Denmark), and cone-beam computed tomography (CBCT) data of a 24-year-old female patient who pursued esthetic treatment with skeletal Class I, mild crowding, and bimaxillary protrusion were obtained. DICOM CBCT data were imported into Mimics (Materialise, Leuven, Belgium) to obtain the root of the dentition model. The reconstructed upper right dentition model derived from CBCT data along with scanned digital cast data was aligned in Geomagic Studio (Geomagic, Morrisville, North Carolina, USA) with the dental crown area, and the dental crown of the plaster model was combined with the dental root from CBCT to form the eventual tooth models. The PDL was simulated with a 0.30-mm shell element [18]. After remeshing, the teeth were aligned to the ideal positions to achieve normal dentition. Each interproximal space between adjacent teeth was set to be less than 0.01 mm. Conventional vertical and horizontal rectangular attachments were designed according to the manufacturer’s suggestion. The CA was developed by making an external offset of dental crowns with a thickness of 0.7 mm [9]. The components of the model are shown in Fig. 1.

For the CA and attachments, the elastic modulus (1,500 MPa, 20,000 MPa) and Poisson’s ratio (0.30, 0.30) were set, respectively [9, 18]. For the PDL, the nonlinear hyperelastic model was used based on the double linear stress‒strain curve of Vollmer’s research: when the dependent variable of PDL ɛ < 7.5%, E1 = 0.05 MPa, and when ɛ > 7.5%, E2 = 0.22 MPa [19]. The dentition model, PDL, attachments, and CAs were assembled in Hypermesh 14.0 software (Altair, Troy, Mich) for further analysis. Unstructured four-node tetrahedral elements were determined for all models. The models were then imported into Abaqus/CAE software (SIMULIA, Providence, RI). Bonded contacts were set between the internal surface of the PDL and roots of teeth and between the external surface of the PDL and alveolar bone. The relationship between aligners and crowns was designated as small-sliding surface-to-surface [20] with the friction coefficient set to 0.2 [21]. Defined after a convergence test, the mesh size was set to 0.20 mm for all models. To simplify the model, only the right half of the maxillary dentition was involved. A contact surface was formed on the midsagittal plane for symmetric analysis [12], and the front end of the CA, on the midsagittal plane, was constrained in the x-direction for symmetric analysis. Normal contact relationship was set between the central incisor and the contact surface. The occlusion plane was defined with the mesial-buccal tips of both upper first molars and the midpoint of central incisors, and the origin of coordinates (the midpoint of central incisors), the X axis (parallel to the line passing mesial-buccal tips of both upper first molars), and Y axis (perpendicular to the X axis) were defined (Fig. 1).

**Fig. 2:**
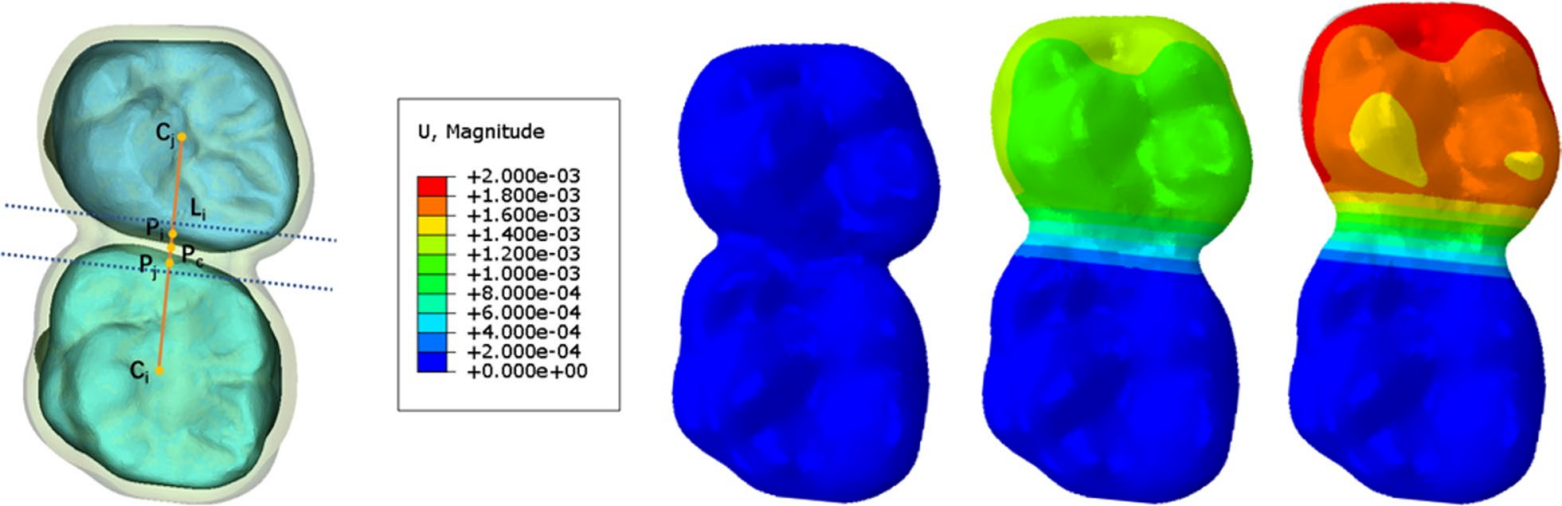
The automatic clear aligner morphology changing method based on the temperature changing method. C_i_–C_j_, the center point of dental crowns at occlusion view; P_i_–P_j_, the margin points of dental crowns on the line of C_i_–C_j_; Pc, the center point of P_i_–P_j_

The temperature changing method (TCM), a common method used in civil engineering and mechanical engineering [22], was used in this study to automatically remodel CAs during long-term orthodontic simulation. As Fig. 2 shows, the center point of dental crowns at the occlusion view was first determined (C_i_, C_j_), the margin points of dental crowns on the line of C_i_–C_j_ were set (P_i_, P_j_), and the center point of P_i_–P_j_ (Pc) was drawn. Perpendicular to the C_i_–C_j_ line, a 1-mm area both mesial and distal from the Pc was determined as the deformation area during staging, and the deformation was restricted to the direction of the C_i_–C_j_ line. The deformation of this area is based on the formula:

$${\text{U}} = {\text{k}}\left( {{\text{d}} + \sum \Delta } \right){\text{t}}$$

where U refers to the preset deformation quantity (0.1 mm per step in this study), k refers to the coefficient of linear expansion, Δ refers to the amount of deformation in previous steps, d refers to the width of the area (2 mm in this case), and t refers to the change in temperature. By determining the above values, the CA at the interdental area can change automatically without the need for manual remeshing (Fig. 2).

**Fig. 3:**
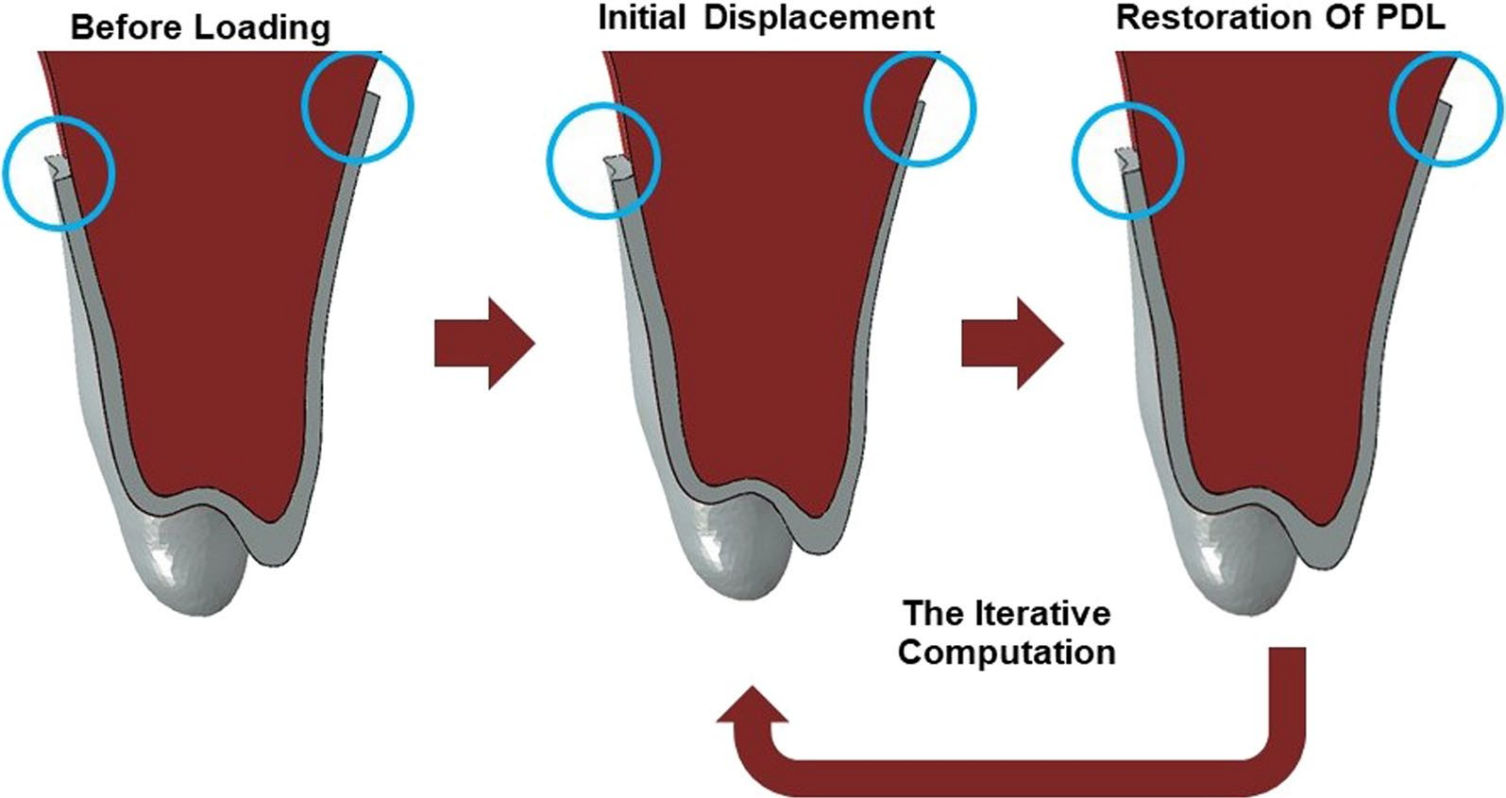
Diagram of the bone remodeling simulation method. Notice the deformation of the periodontal ligament in the blue circles. PDL, periodontal ligament

Based on the bone remodeling simulation method revealed by Hamanaka et al. [12], the tooth and the alveolar bone were assumed to be rigid bodies during the initial displacement since their deformation is negligible compared with that of the PDL. During the 2 phases of bone remodeling simulation, the outer surface of the PDL was constrained, and the tooth moved under orthodontic forces with the strain of the PDL. Then, in the second phase, the tooth position was reserved for further calculation, and the PDL was restored to its original configuration and thickness. These 2-step calculations were iterated to simulate orthodontic tooth movement after the long-term process (Fig. 3).

In this study, 2 weeks of CA wearing during staging could be simulated with 2 iterative computations of PDL combined with 1 iteration of CA remodeling. Before each iteration, the CA in the previous step was deleted and regenerated, and a best fit algorithm was performed for the inner surface of the CA and dental crowns to simulate the wear-in process. These procedures were executed automatically using custom-developed subroutines with Python for ABAQUS.

In this study, a prescribed 2-mm distalization of the second molar was simulated. As a previous study suggested, tooth movement as rigid-body motion can be described as a combination of translation and rotational motion around an axis of rotation and a constant point that is fixed in the tooth, such as the long axis (LA) of the tooth and the center of resistance point (CR) [23]. The crown point was set at the midpoint of the incisor edge, the cusp of the canine, and the midpoint of the occlusion surface of the premolars and molars. The root point was set as the apex of the root of single-root teeth and the apex of the mesial root of multirooted teeth. The CR was determined as the midpoint of the section at the 1/3 root level for single-root teeth and the midpoint of the section at 1 mm above the furcation point for multirooted teeth. LA was determined as the line of crown point and CR for each tooth. The 3D displacement of the crown points and root points and the rotation of the LA at each step were recorded. The distance between the distal cusp points of the second molar and the corresponding CA inner surface points were measured to evaluate the offtrack. The efficacy of the second molar distalization and the interdental space opening between molars at each step were calculated by the actual distal displacement of the second molar and the enlargement of the distance between the crown points of the two molars divided by the prescribed movements, respectively.

## Results

**Fig. 4:**
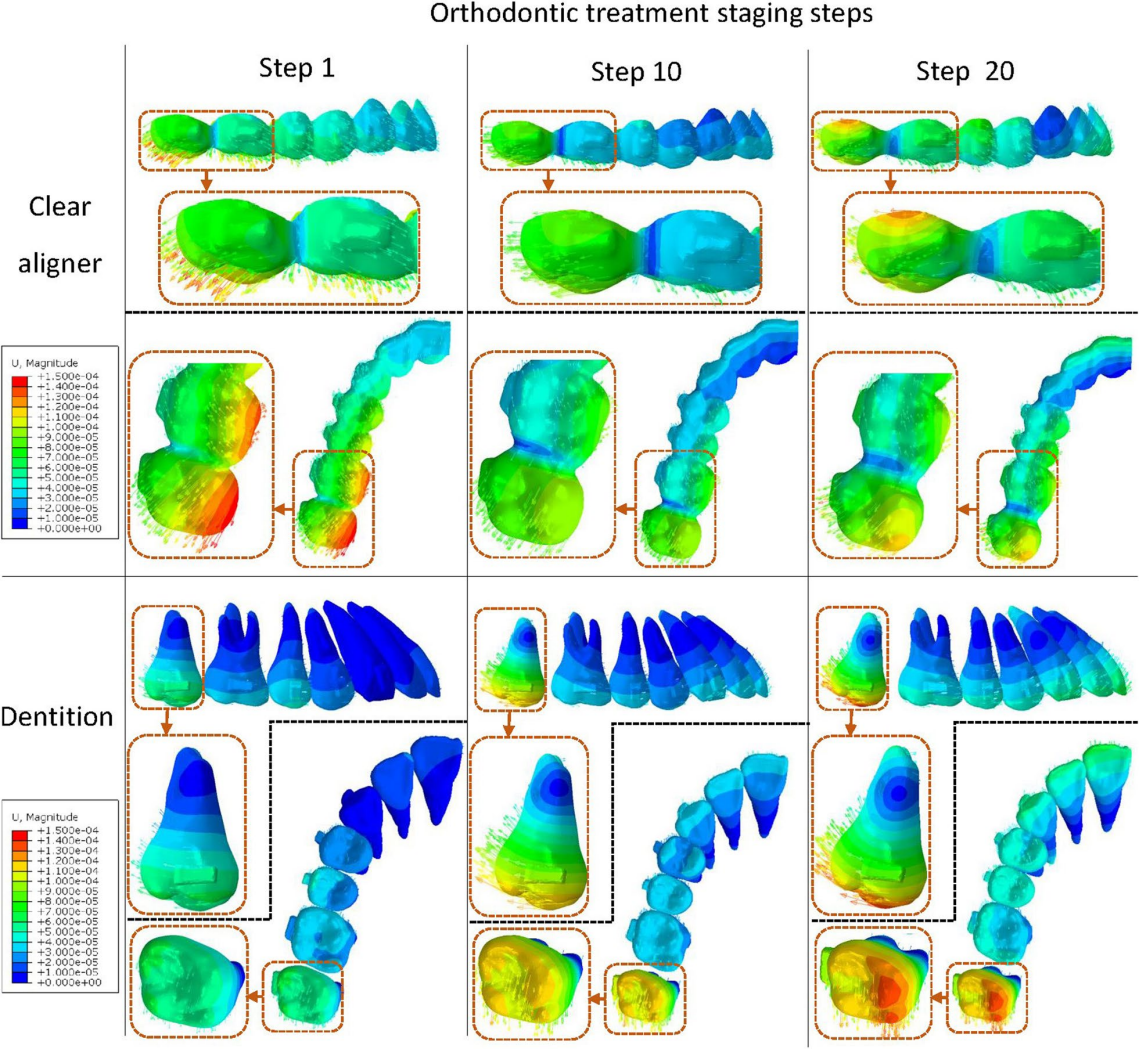
Teeth and clear aligner displacement at different treatment staging steps

**Fig. 5:**
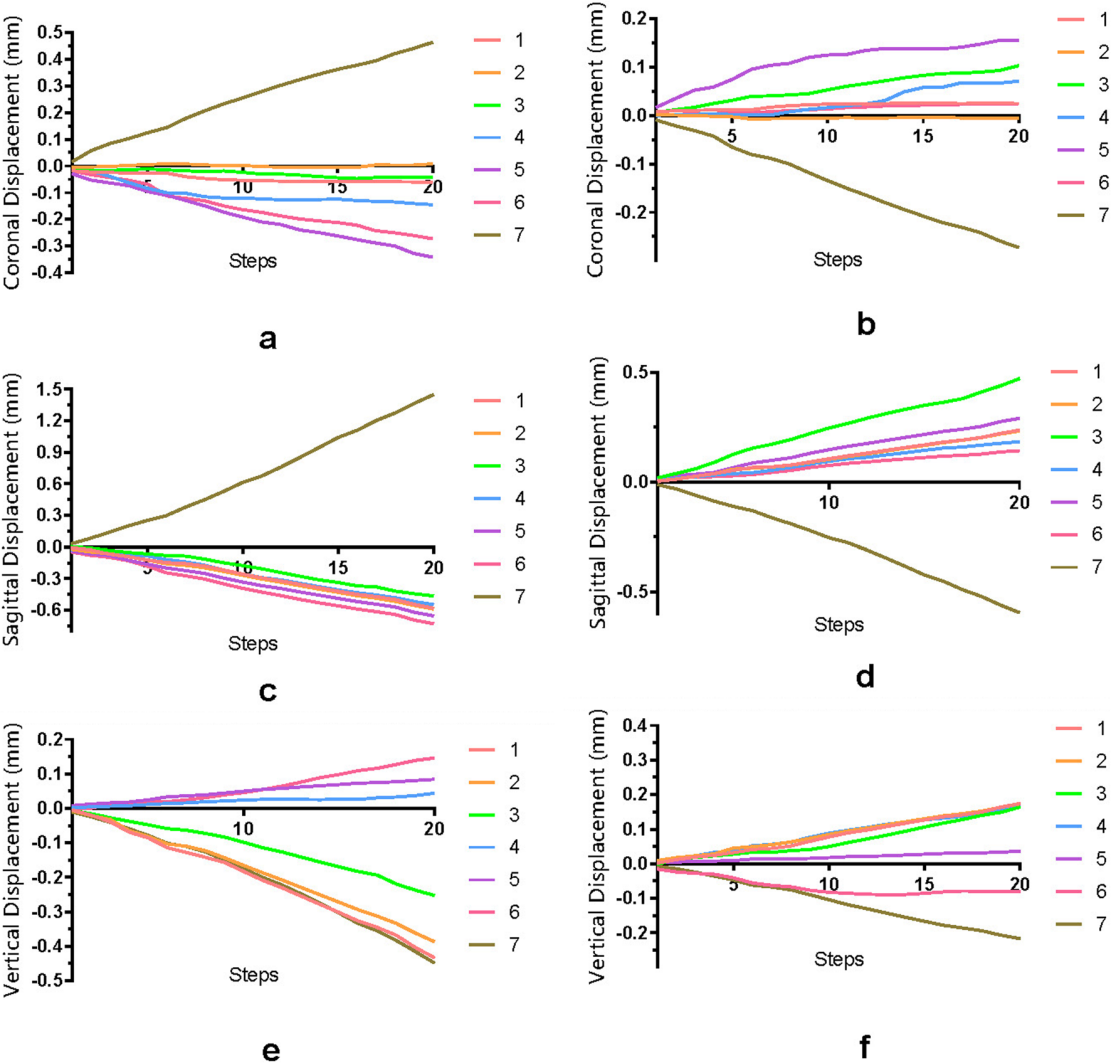
Three-dimensional displacement of the crown points (**a**, **c**, **e**) and the root points (**b**, **d**, **f**) of each tooth. Tooth numbering according to the FDI tooth numbering system

**Fig. 6:**
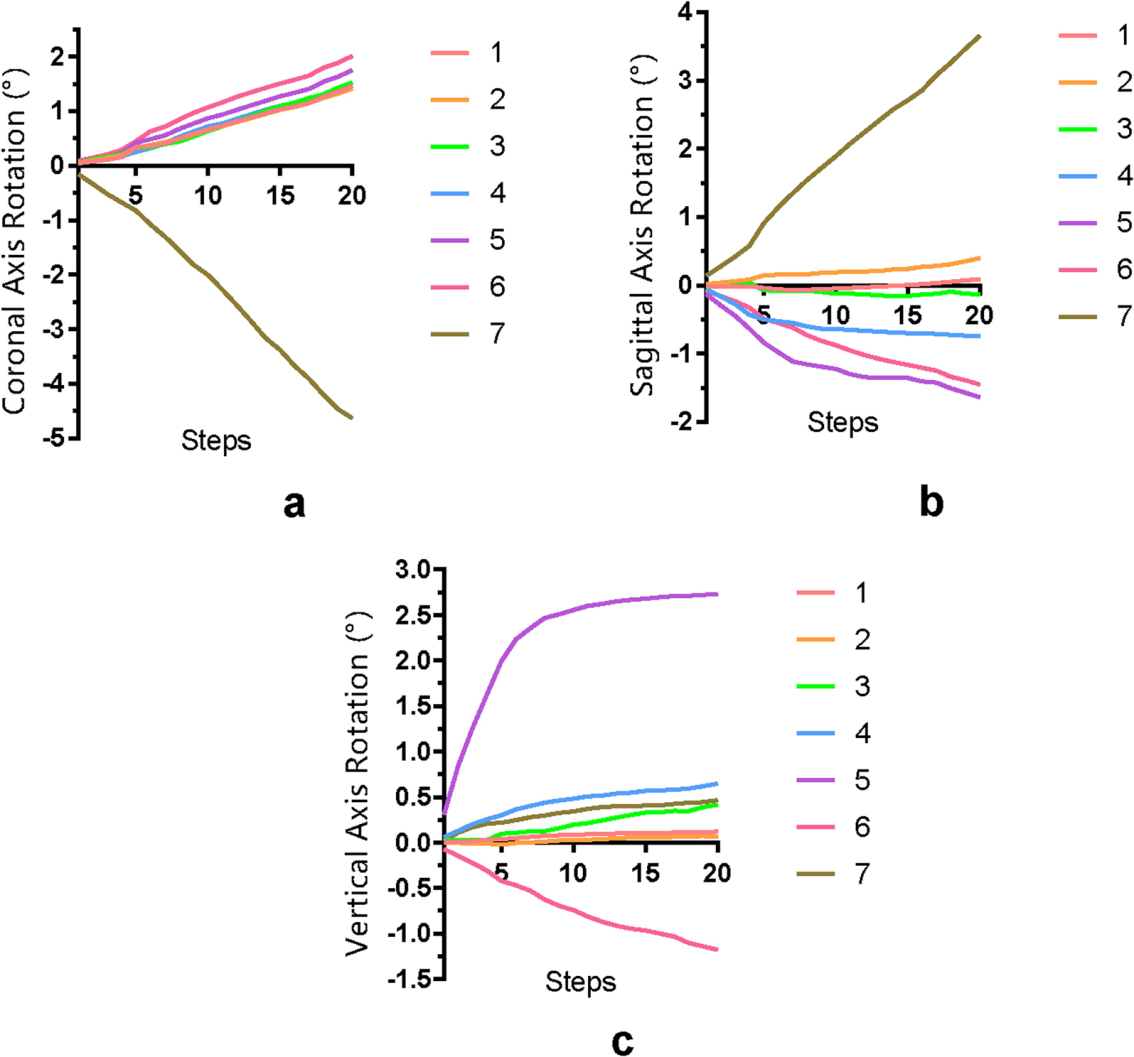
Three-dimensional rotation of the long axis of each tooth: coronal axis rotation (**a**), sagittal axis rotation (**b**), and vertical axis rotation (**c**). Tooth numbering according to the FDI tooth numbering system. The positive axis was determined by the right-handed rule

As shown in Fig. 4, the prescribed 2-mm distalization of the second molar was simulated. Along with the staging progress, in addition to the obvious stretching of CA between molars, the occlusal displacement tendencies of CA at molars were revealed from the lateral view. Distal and buccal displacement of the second molar and the corresponding CA area was noticed. Significant distal tilting of the second molar was revealed, with the center of rotation at approximately around the apical third. Moreover, mesial and slight occlusal displacement of bicuspids and first molar was noticed, along with the proclination of anterior teeth, which caused the ‘reversed bow effect’. The 3D displacement of the crown points and the root points of each tooth during different staging processes was quantified and is shown in Fig. 5. For coronal displacement, buccal displacements of the crown of the second molar were observed (0.46 mm at step 20). Both incisors and canine showed slight coronal displacement less than 0.1 mm. All the other teeth showed lingual displacement, with the greatest displacement of the second premolar (0.34 mm at step 20). For the sagittal displacement, for the second molar, significant distal displacement of the crown (1.45 mm at step 20) and mesial displacement of the root (−0.60 mm at step 20) were revealed. During staging, intrusion of the anterior teeth and the second molar was noticed. The intrusion of the central incisor, the lateral incisor, and the second molar was 0.43 mm, 0.39 mm, and 0.45 mm, respectively, at step 20. The 3D rotation of the long axis of each tooth was quantified and is shown in Fig. 6. The results of rotation of the tooth axis along the coronal axis, i.e., mesiodistal rotation of posterior teeth and labiolingual rotation of anterior teeth, showed −4.63°distal tilting of the second molar at the 20th steps. All the other posterior teeth showed mesial tilting, and all the anterior teeth showed proclination of approximately 1.41°–2.01° at the 20th step. In addition, 3.65° buccal rotation and 0.47° distal tilting of the second molar were revealed at the 20th step. Lingual rotation was noticed from the canine to the first molar. In addition to the second molar, the second premolar gained the largest lingual and distal rotation (− 1.64° and 2.73° at step 20). A mesial rotation tendency was noticed for the first molar (− 1.18° at step 20).

**Fig. 7:**
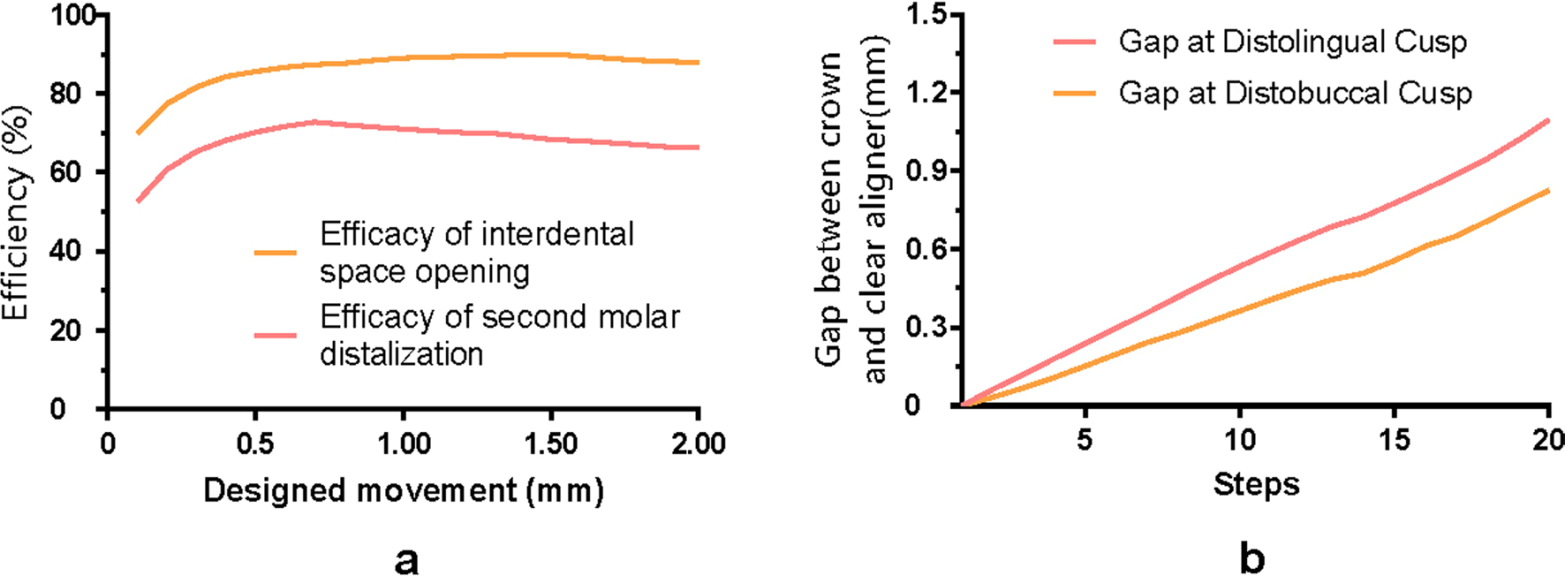
Efficacy of second molar distalization and interdental space opening between molars (**a**). The distance between the distal cusp points of the second molar and the corresponding CA inner surface points (**b**)

The efficacy of second molar distalization first increased and then decreased, with a peak of 72.8% at the 7^th^ step (Fig. 7a). The average efficacy was above 67% during the prescribed 2-mm molar distalization (Table 1). Likewise, the same tendency was noticed for the interdental space opening between molars, with a peak of 90.01% at the 15^th^ step. The average efficacy was highest (89.12%) during the 1–2-mm designed movement. As for the offtrack at the second molar (Fig. 7b), both gaps at distolingual or distobuccal cusps increased with steps, which were both less than 1.1 mm at step 20. Gap at the distolingual cusp was slightly larger in each step.

**Table 1:** Average efficacy of second molar distalization and interdental space opening between molars

	Designed movement			
	0–0.5 mm	0.5–1 mm	1–1.5 mm	1.5–2 mm
Average efficacy of second molar distalization (%)	63.45 ± 7.02	71.83 ± 0.65	69.65 ± 0.85	67.21 ± 0.61
Average efficacy of interdental space opening (%)	79.82 ± 6.37	87.84 ± 0.92	89.67 ± 0.31	88.58 ± 0.69

## Discussion

Currently, all published studies investigating CA with FEM were limited to the initial status, which could provide results with limited clinical significance. In this study, a time-dependent FEM that enables the long-term analysis of orthodontic tooth movement with CAs was developed. However, this paper focused on the illustration of the novel 4D FEM method, no comparisons among different anchorage designs were considered. According to the results, during the molar distalization, the flared anterior teeth emphasized the necessity of anchorage augmentation, and the thickness of labial side alveolar bone at anterior teeth should be evaluated before the movement design. The distal tilting for the second molar called for further better attachment design.

To carry out ‘4D’ FEM, which takes the biomechanical response as another dimension, researchers have used various iterative computation methods to simulate this iterative biomechanical behavior [11–15]. However, in most of these studies, the tedious time-consuming procedures and the remeshing requirement of the model after each iteration restricted the usage of the methods. This study used the bone remodeling simulation method revealed by Hamanaka et al. [12]. This simulation method omits the need to model the alveolar bone, which saves time and is easy to converge during multiple-step simulation. However, as the authors pointed out, the iteration steps of this simulation method cannot match the clinical treatment time.

Previous studies reported that tooth movement remained steady at approximately 0.2–0.3 mm/week when the strain in the PDL reached above 0.3% [11, 24]. Meanwhile, the average PDL thickness was 0.15–0.38 mm, and it was commonly presumed to be 0.2 mm or 0.3 mm in previous FEM studies [12, 25], which was approximately the same value as the maximum weekly tooth movement during orthodontic treatment. Thus, by using the iterative computation simulation method revealed by Hamanaka et al. [12], we presumed that each iteration refers to 1 week of tooth movement, and the automatic staging of CA can be further combined.

TCM is based on the principle that heat causes an object to expand cold causes an object to contract. By determining the expansion/shrinkage coefficient, change in temperature, and expansion direction, the CA at the interdental area can change automatically with high accuracy and without the need for manual remeshing, which makes the iterative morphology change of CA possible.

During molar distalization, the ‘reversed bow effect’, which leads to flared anterior teeth causing deep overjet and shallow overbite, was noticed. Similar to the results of this study, Ravera et al. [5] revealed an average of 1–4° proclination of middle incisors during molar distalization. Likewise, Caruso et al. [6] noticed an average of 2–11° of distal tilting and 1–2-mm intrusion of molar distalization with CA. However, less intrusion of the second molar was found in this study, which may be due to the occlusal pad effect of CA during the clinical treatment that enlarges the intrusion. The results of this study showed 4.63° distal tilting for the second molar with 2 mm of prescribed distalization. Recently, Badr et al. [26] investigated 38 patients treated with Invisalign with a molar distalization design. The findings revealed that most teeth affected by anchorage loss were central incisors, followed by lateral incisors and then canines. Regarding the results of this study, a similar proclination of both incisors and canines was noticed during molar distalization.

In our study, the expression rate of the designed distalization of the second molar was relatively low (approximately 68%) compared to the high efficacy of interdental space opening between molars with CA (approximately 89%). By taking the labial displacement and inclination of anterior teeth into consideration together, which indicated anchorage loss of anterior teeth, the necessity of anchorage augmentation during molar distalization should be emphasized. Simon et al. [1] reported an expression rate of 88% for molars when a mean distalization movement of 2.7 mm was prescribed. However, the study only used a dental model for superposition and measurements, the accuracy of which was open to doubt. Recently, Badr et al. [26] revealed that the overall efficiency of maxillary molar distal movement produced by CAs amounted to 73.8%, with that of the first molar (75.5%) being slightly higher than that of the second molar (72.2%), which was similar to the results (68%) of this study.

However, the limitations of this study are also obvious. First, during clinical treatment, for most cases, mid-course correction was immediately prescribed once significant offtrack was observed. The occlusal pad effect of CA also contributes to the fitness of CA, which was not simulated in this study. Additionally, the proof and improvement of the accuracy of the iterative computation methods of tooth-movement simulation used in this study need further clinical evidence. Moreover, with the current bone remodeling simulation method, individual factors, such as the bone density, the cortical bone boundary, and the different response levels to orthodontic force, which may interfere with the distalization of molars, have not been considered.

## Conclusion

1. A time-dependent FEM that enables the prediction of orthodontic tooth movement during molar distalization with CAs was developed. With the TCM, the morphologic changes of CAs during staging were successfully simulated.
2. The FEM results suggested distal tilting of the second molar and the proclination of anterior teeth during molar distalization. The expression rate of the designed distalization of the second molar was approximately 68% for 1–2-mm prescribed molar distalization without anchorage augmentation.

## Data Availability

Not applicable.
